# Next-Generation Psychedelic Drug Development

**DOI:** 10.1021/acschemneuro.6c00492

**Published:** 2026-09-14

**Authors:** Lindsay P. Cameron, Matthew W. Johnson, Kevin S. Murnane, Mark M. Rasenick, Charles D. Nichols

**Affiliations:** † Nancy Pritzker Laboratory, Department of Psychiatry and Behavioral Sciences, 6429 Stanford University, Stanford, California 94305, United States; ‡ Center of Excellence for Psilocybin Research and Treatment, Sheppard Pratt, Baltimore, Maryland 21204, United States; § Department of Pharmacology, Toxicology & Neuroscience, School of Graduate Studies, Louisiana State University Health Shreveport, Shreveport, Louisiana 71103, United States; ∥ Louisiana Addiction Research Center, Louisiana State University Health Shreveport, Shreveport, Louisiana 71103, United States; ⊥ Department of Psychiatry and Behavioral Medicine, School of Medicine, Louisiana State University Health Shreveport, Shreveport, Louisiana 71103, United States; # Departments of Physiology & Biophysics and Psychiatry, University of Illinois College of Medicine, Chicago, Illinois 60612, United States; ∇ Jesse Brown VA Medical Center, Chicago, Illinois 60612, United States; ○ Pax Neuroscience, Glenview, Illinois 60015, United States; ◆ Department of Pharmacology and Experimental Therapeutics, 23346 Louisiana State University Health Sciences Center, New Orleans, Louisiana 70112, United States

**Keywords:** psychedelics, psilocybin, nonpsychedelic analogs, 5-HT2A, Biotech, drug development

## Abstract

Psychedelic medicine has gained rapid popularity for the treatment of a variety of neuropsychiatric disorders, including depression, anxiety, and post-traumatic stress disorder. As novel psychedelic analogues and treatment regimes emerge, increasing numbers of variables are being tested. Here, we review various approaches and considerations for novel psychedelic therapies, including the strengths and limitations of current assays and models. Finally, we provide guidance and considerations for next-generation psychedelic drug development.

## Graphical abstract

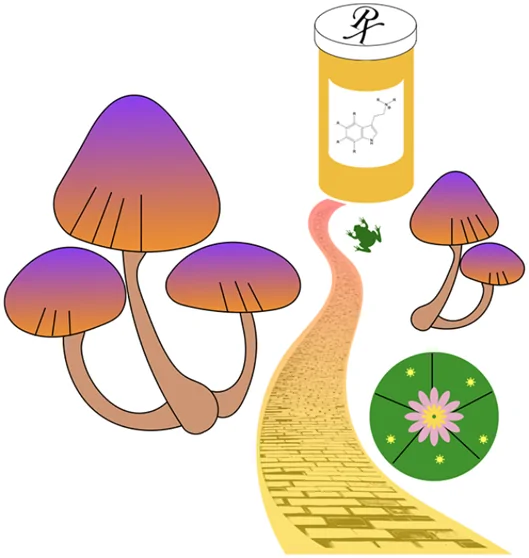

## Introduction

Psychedelic compounds have taken center stage in modern neuropsychiatric research. Psilocybin is found naturally occurring in dozens of species of mushroom and has shown remarkable efficacy in clinical trials for major depressive disorder,^1,2^ cancer-related distress,^3,4^ alcohol use disorder,^5^ tobacco use disorder,^6^ demoralization in AIDS survivors, and bipolar II disorder.^7^ Its therapeutic effects are mediated by its active metabolite psilocin, which is a nonselective serotonin receptor agonist and psychedelic.^8^ Moving beyond psychiatry, promising recent research has been conducted in the field of neurology with positive findings for psilocybin in the treatment of migraine and cluster headache disorders.^9^ The findings for neuropsychiatric disorders are notable for several reasons. First is the observation that a high percentage of patients are helped. Response rates demonstrate remission from these neuropsychiatric disorders in most participants, and clinically significant improvement in many participants. The second is the persisting nature of such improvements. Such results are often observed several months or even more than a year after treatment. Third, such results are achieved after only a small number of medication administrations, typically one, two, or three administrations of the psychedelic compound. Although initial preparatory meetings and follow-up discussion (“integration”) sessions are conducted, this is still a remarkably small number of administrations.

Despite this incredible promise, there are several major limitations to these treatments that will pose barriers to widespread clinical application upon potential approval by the United States Food and Drug Administration, the European Medicine Agency, and other regulatory bodies. Other barriers to adoption of psychedelic therapy involve both difficulties in the current administration model as well as the potential for insurance and government payer agencies to approve payment for what is now poised to be a very expensive treatment with substantial professional time. The serotonin-releasing agent 3,4-methylenedioxymethamphetamine (MDMA; an entactogen, but with some overlapping effects with psychedelics) has shown strong efficacy in the treatment of post-traumatic stress disorder (PTSD).^10^ Recent trials investigating MDMA for PTSD cost $11,537 per patient, and psychedelic trials will likely be within this range as well.^11^ The majority of this cost comes from paying medical professionals for the duration of therapy and dosing sessions. Given that patients who often need these mental health therapies have lower socioeconomic status, this cost—not to mention the travel costs, many days off work for several weeks of sessions, childcare, etc.—is not currently conducive to creating an accessible treatment. However, psychedelic-inspired medications and novel molecular entities may hold promise to address many of these concerns.

One common definition of a “psychedelic” is a compound that elicits alterations in perception, cognition, emotions, and sense of self via activation of 5-HT_2A_R. For the purpose of this review, we employ another commonly used definition of “psychedelic” that broadly describes compounds that elicit such effects regardless of pharmacological site of action. Here, we discuss several drawbacks to first-generation psychedelic and related compounds currently under investigation (e.g., psilocybin, MDMA), and target properties of novel compounds that may address these limitations.

This Review will attempt to coalesce recent advances in psychedelics from the standpoint of both research and therapy. The authors also wish to discuss current impediments and propose potential solutions that might establish a rationale for psychedelic therapy and chart a path forward for those who hope to bring it to fruition.

### Nonpsychoactive Analogs

First-generation psychedelics [e.g., psilocybin, lysergic acid diethylamide (LSD), *N,N*-dimethyltryptamine (DMT), 5-methoxy-*N–N*-dimethyltryptamine (5-MeO–DMT), mescaline] are known for their dramatic subjective effects (Figure 1). Indeed, it is difficult to overstate the magnitude with which patients often describe such experiences, and how unique and enduring these effects can be. For example, the construct of “mystical experience” has been used in the assessment of psychedelic effects. Such effects involve strong endorsement of elements including senses of unity (e.g., feeling at one with the universe), timelessness, spacelessness, ineffability, and positive mood.^12,13^ The construct of mystical experience is highly correlated with (and might be identical with) the construct of “oceanic boundlessness.”^14^ By definition, these are not everyday experiences that are easily characterized. This relates to the often-debated notion and observation that it is difficult to conduct credible “blinded” research (in which the participant is kept uninformed as to whether he/she received an active drug or placebo).^15^ Notably, nonpsychoactive analogs would be able to circumvent these important blinding confounds that first-generation psychedelics face. Important for the current discussion is the debate about whether subjective effects are necessary for therapeutic effects.^16^ On one hand, some studies have shown a relationship between the intensity of subjective effects on the psychedelic administration session day with long-term therapeutic outcomes.^12^ On the other hand, a growing number of studies have shown in nonhuman animals that analogs of first-generation psychedelic compounds that may lack or minimize subjective psychoactivity lead to multiple forms of neuroplasticity,^17–19^ with experimental evidence suggesting postacute therapeutic-type effects in nonhuman behavioral models.^18,20,21^ The use of animal models for studying psychedelic action and neuroplasticity is described below.

**Figure 1:**
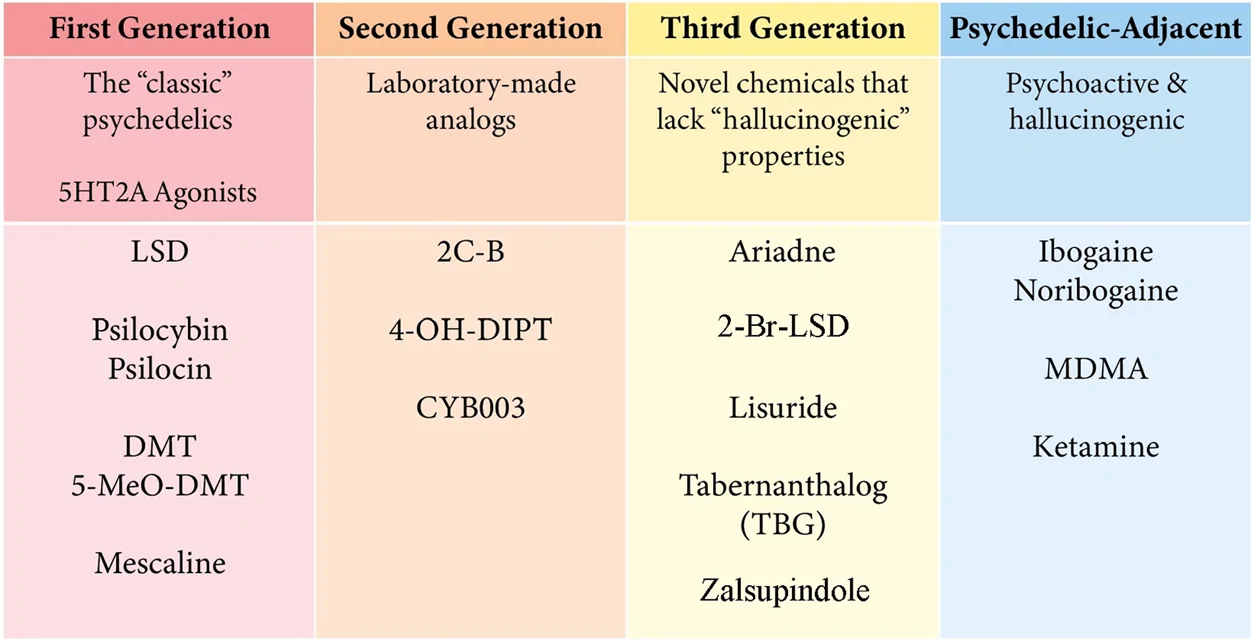
Description of generations of 5-HT_2A_ receptor agonists, and examples from each class.

We do not have direct evidence currently regarding whether such neuroplastic effects occur in humans and, if they do, whether they contribute to therapeutic effects. Clearly, psychedelic therapy can result in behavioral plasticity by definition in that we observe behavioral change (therapeutic learning). We believe that such neuroplastic effects are at least one component of what might be multiple mechanisms that underlie the therapeutic potential of first-generation psychedelics. There is likely some false dichotomy in debates about whether subjective effects are necessary for therapeutic effects. For example, subjective experiences and neuroplastic effects might mediate first-generation psychedelic therapies. In this vein, nonpsychoactive second-generation analogues of psychedelics, while not requiring psychoactive effects, could be inducing similar levels of neuroplastic changes through differing mechanisms that result in therapeutic effects. For example, experience-dependent plasticity may be a component of classic psychedelic therapeutic mechanism of action in human patients, but this would be lacking with nonbehavioral analogues. If this were the case, then it may be conceivable that such analogues might require different treatment models. These may include more regular dosing (e.g., every few weeks as needed or else daily dosing as with typical psychiatric medications). If patients were able to self-administer these compounds and continue their daily lives, then this would drastically increase the accessibility of these medicines. Or, it might be optimal to explicitly pair the administration of such compounds to learning trials or therapeutic procedures, as has been explored with the NMDA agonist, d-cycloserine, in the facilitation of time-limited extinction-based therapies for phobias and other anxiety disorders.^22^ There are, unfortunately, limited clinical data in humans for nonbehavioral analogues of psychedelics in the treatment of neuropsychiatric disorders. Only one clinical trial has been performed to date with a nonbehavioral psychoplastogen, zalsupindole, by Delix Therapeutics, which showed sustained therapeutic effects in an MDD phase 1b clinical trial after only one treatment.^23^ Further research needs to be performed, however, to fully understand the potential therapeutic efficacy of nonbehavioral analogues for factors like dosing and efficacy, which may be compound-specific.

Given the potential for nonpsychoactive analog compounds, it would be desirable to create second- and third-generation analogs that are both maximally and selectively neuroplastic (Figure 1). If neuroplasticity underlies psychedelics and psychedelic analogs’ therapeutic effects, then it is unknown what form or forms of neuroplasticity are important (for example, dendritic branching versus synaptogenesis), and which human brain regions are most affected. Nonpsychoactive compounds (third-generation analogs) could leverage improved information on the therapeutically functional form or forms of neuroplasticity, and parametric information on the degree and timing of such effects. Furthermore, it can increase accessibility for patients, decrease cost, and minimize adverse events such as having to deal with difficult experiences. Finally, many individuals would prefer to minimize altered experiences.

### Duration of Psychoactive Effects

Regardless of whether nonpsychoactive compounds have ultimate therapeutic potential, the best current evidence based on clinical trials is for overtly psychoactive compounds incorporated into the “psychedelic therapy” model. Rather than referring to any therapeutic use of psychedelics, this term refers to a specific model in which relatively high doses of psychedelics in patients who have been prepared for the experience, have developed a rapport with the professional support personnel who will be present with them during the psychedelic session, and follow (“integration”) sessions are conducted in which the experience is discussed in the days following the psychedelic administration session. Patients are encouraged to pay attention to their experience during the psychedelic administration session and to minimize attention to the external environment, often with the use of eyeshades and supportive music during the experience. This model has roots that date back to the late 1950s.^24^ It seems likely that this model will continue to be used given the strong current evidence, and given that long-term therapeutic benefits seem to be related to lessons learned during or in the aftermath of the subjective experience.^25,26^ Although the psychotherapy-coupled model predominates, there is evidence from several clinical studies that psychedelics can produce similar therapeutic efficacy in patients without receiving psychotherapy sessions. Among these are LSD for generalized anxiety and 5-MeO–DMT for treatment-resistant depression trials, which demonstrated therapeutic efficacy.^27,28^

Despite the apparent therapeutic responses to high-dose overt psychedelic therapy, the duration of effects is likely a clinical barrier that might minimize clinical utilization. As previously outlined, the current treatment model is extremely expensive and prohibits access. While cost-effectiveness analyses might show that such an expense is warranted if long-term results are highly efficacious, it may be a challenge for obtaining payor coverage for such treatments in countries like the United States. Regardless of payer coverage, fewer individuals might be willing to engage in such lengthy treatment sessions. While we still need empirical research to determine if long-acting compounds like lysergic acid diethylamide (LSD) might show improved or similar efficacy to moderate-duration compounds such as psilocybin and MDMA, the fact that psilocybin and MDMA are more common in modern research compared to LSD (more commonly used in the 1950s–1970s research) represents at least some improvement regarding this concern. However, there is substantial current commercial and clinical interest in compounds that are even shorter-acting than psilocybin and MDMA, which last approximately 6 and 4 h, respectively. The psychedelic chemist Alexander Shulgin, for example, expressed interest in the therapeutic potential of 4-HO–DIPT, a classic psychedelic with a duration of 2–3 h, for use in psychotherapy.^29^ Similarly, there is substantial interest in the very short-acting classic psychedelics, DMT and 5-MeO–DMT.^30–32^ Investigation of not only these but also novel second- and third-generation compounds, either related to classic psychedelics or to serotonin-releasing compounds similar to MDMA, yet with shorter durations of action. Will such short-acting compounds show similar efficacy to longer-lasting compounds? How might varying subjective experiences provided by different drugs affect therapeutic outcome? These are empirical questions that need investigation. Though compounds such as LSD and psilocybin appear effective, shorter-duration psychedelic compounds may greatly expand access to psychedelic therapeutics.

### Challenging Experiences

Challenging experiences (known colloquially as “bad trips”) appear to occur occasionally with classic psychedelic compounds such as psilocybin and LSD.^33,34^ To date, these appear manageable in the vast majority of cases, and there is some evidence that they may even be clinically beneficial for some patients.^33^ Nonetheless, these adverse reactions present the potential for problematic occurrences such as extreme paranoia or confusion leading to accidents. Just because such potentially dangerous outcomes have not occurred in the highly managed trials of academic research today does not mean that they will not occur when widely clinically available and managed by less highly trained clinicians.

One goal of third-generation psychedelic drug development, therefore, would be to devise compounds with substantially lower potential for challenging experiences while maintaining aspects of subjective effects showing a relationship to therapeutic outcomes such as mystical experiences. In some sense, MDMA represents such an example. While MDMA experiences can be difficult, particularly when addressing trauma, such challenging experiences might be more likely to involve difficult emotions, such as those involved with remembering and processing the traumatic experiences and the negative self-realization that can result from trauma. MDMA is less likely to lead to anxiety, paranoia, and derealization that can occur with classic psychedelics, such as psilocybin.^35,36^ However, mystical experience tends to occur more frequently and with greater profundity with classic (first-generation) psychedelics.^37,38^ Whether the potential for intense mystical experience scores is inherently tied to an increased likelihood of challenging experiences is an open empirical question. Current studies are investigating the use of psilocybin or LSD with MDMA as a means to neutralize these undesirable experiences.^39^ Therefore, the development of compounds with high potential for mystical experience with very low potential for challenging experience is a very worthwhile area for investigation. Overall, the goal would be to create compounds that result in sessions that are maximally manageable under a broader range of conditions. Notably, nonpsychoactive analogues of psychedelics would circumvent the necessity to prevent challenging experiences.

### Reliability of Experience

The increased reliability of mystical experience is an important area of investigation for second-generation psychedelics. For novel compounds that strive to retain hallucinogenic properties, maximizing the number of patients who find their psychedelic experiences to be personally meaningful and insightful is an important goal. Although maximally supportive conditions produce positive experiences, these typically occur in most patients in high-dose therapy with classic psychedelics; they do not occur in all participants.^12^ Furthermore, it is possible that nonresponsive patients have worsening symptoms following unsuccessful treatment, which is often attributed to their “last resort” not working. Increasing the rates of these effects at physiologically safe doses would therefore be an important goal of second-generation psychedelic drug development. Further, it is likely that placebo-controlled (or active comparator) double-blind studies will be required, though fully effective blinding in such studies is difficult to conduct.^15^

### Physiological Safety

Aside from increasing safety by making the psychoactive effects more manageable, safety can be increased by increasing the physiological safety of the first-generation compounds. Although classic psychedelics such as psilocybin are remarkable in their relative physiological safety,^40^ they are not without safety concerns. Because classical psychedelics modestly and transiently increase blood pressure and heart rate, they can be a risk factor for individuals who are cardiovascularly compromised. Indeed, modern clinical trials exclude such individuals, and potential postapproval clinical use of these compounds is likely to maintain the same exclusion of such individuals. Therefore, the development of second- and third-generation psychedelic compounds with less cardiovascular impact would allow for the expansion of the treatment. This is especially important when considering the evidence for psilocybin in the treatment of seriously ill cancer patients, many of whom have compromised cardiovascular, respiratory, and metabolic function. While MDMA also appears to be safe for healthy patients, it has greater acute cardiovascular risk than classic psychedelics such as psilocybin and LSD. Therefore, the development of second-generation MDMA-like compounds with a safer cardiovascular profile would enable the treatment of a larger patient population.

Another domain for potential physiological concern for first-generation psychedelic compounds regards the agonist activity that major psychedelics, including psilocybin and LSD, or indirectly like MDMA, produce at 5-HT_2B_ receptors.^41^ Agonist activity at this receptor has been linked mechanistically and empirically to valvular heart disease. It was this activity and mechanism that led to the Food and Drug Administration withdrawal of clinical approval for the diet medication Fen-Phen in the 1990s. While it would appear that current treatment models that only administer one to three doses of the psychedelic might not represent clinically relevant concern for such toxicity, there is a requirement to explore the need for repeated dosing, especially for treatment-resistant disorders or for the frequent administration of minimally psychoactive doses (colloquially known as “microdosing”).^42^ While empirical work might ultimately allay concerns about heart valve damage with psychedelics, these are currently unaddressed empirical questions that may be parametrically dependent. Therefore, in the development of second-generation compounds, the elimination or minimization of agonist activity at 5-HT_2B_ receptors is an important goal.

In the same vein, other compounds used in psychedelic therapy such as ibogaine inhibit hERG channels in the heart, potentially leading to arrhythmias and heart attacks.^43^ This is a common property of many lipophilic drugs; therefore, current novel psychedelic synthesis strategies have (and should continue to) minimize interactions with hERG. While one possible approach is using coadministration of magnesium to mitigate this risk,^44^ newer-generation analogues lacking this form of toxicity should be investigated.

Other 5-HT receptors may be involved in psychedelics’ unwanted effects. 5-HT_1A_ receptors may be involved for tryptamine and ergoline psychedelics because those particular chemotypes show affinity and efficacy for nearly all serotonin receptors. Further, ergolines like LSD can have appreciable affinity and efficacy at all monoaminergic GPCRs, particularly dopamine receptors, in addition to 5-HT receptors.^45^ Analogs lacking these properties would be beneficial. Figure 2 illustrates several of the common targets of various psychedelic drugs, and Table 1 shows the Emax data for each receptor used to generate Figure 2.

**Figure 2:**
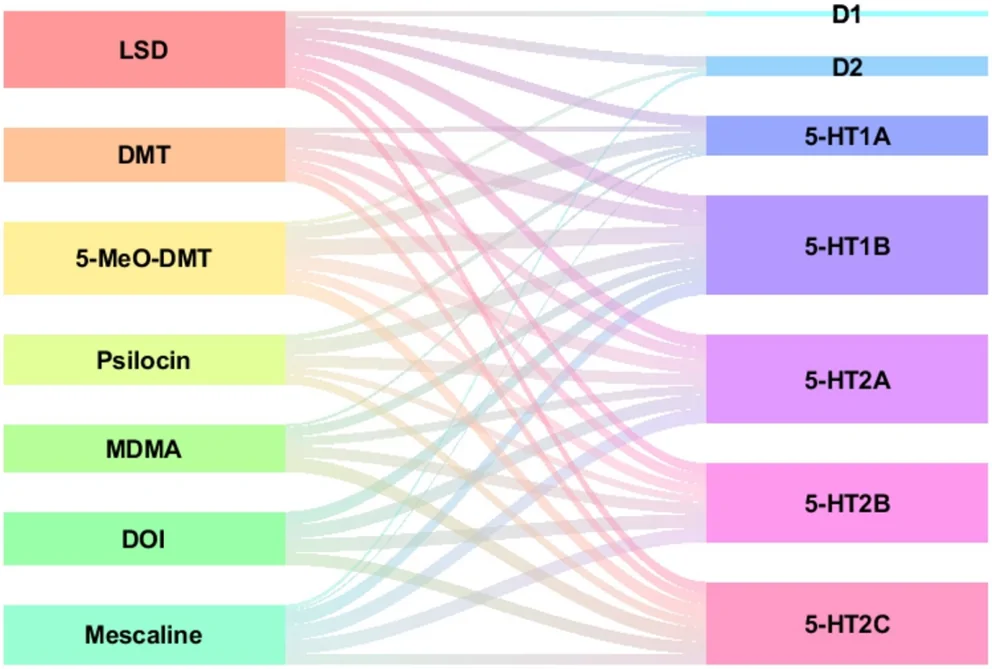
Schematic of psychedelic drug activity at common receptor targets. Efficacy values (Emax) used to generate this schematic are presented in Table 1.

**Table 1:** Emax Values of Psychedelics at Various Important CNS Receptors (Reported as Percent of 5-HT Stimulation)^a^

	D1	D2	5-HT1A	5-HT1B	5-HT2A	5-HT2B	5-HT2C
LSD	136.6 ^155^	82 ^66^	102.16 ^76^	100 ^156^	76.5 ^76^	70 ^157^	29 ^157^
DMT			93.5 ^76^		66.02 ^76^		103.15 ^158^
5-MeO–DMT			107 ^76^	86.8 ^60^	83.25 ^76^	66 ^60^	100.5 ^158^
Psilocin			58.31 ^76^		50.77 ^76^		103.77 ^158^
MDMA					70.41 ^158^		195.65 ^158^
DOI				108 ^158^	99.32 ^65^	103.2 ^65^	114.3 ^65^
Mescaline			22.22 ^76^		81.15 ^76^	91.1 ^65^	108.5 ^65^

a References here for Figure 1.

### Approaches to Development of Next Gen Drugs

Due to the lack of perceived medicinal value of psychedelics, and their scheduling as dangerous substances by the DEA, there was very little interest by the scientific community in general to develop new agonist ligands for 5-HT_2A_ receptors after 1970. There were two notable exceptions: Dr. Alexander Shulgin and Dr. David E. Nichols. Dr. Shulgin began his chemical experimentation with psychedelic chemicals in the 1960s, with several years of experience from Dow Chemical under his belt. He is credited for the (re)­discovery of MDMA’s behavioral effects and the development of certain phenethylamine drugs in the 1960s. Throughout his lifetime, he created hundreds of second-generation psychedelics based upon the classic psychedelics psilocybin, mescaline, and LSD. He had a DEA license for analytical research to synthesize novel chemicals that he used primarily to make substituents and analogs of psychedelics. However, he often sampled the compounds himself in risky self-experimentation that generally lacked scientific rigor. Based on his impressions of the effects, he then further generated additional variants of molecules that he found interesting. Although he published many of his syntheses in peer-reviewed journals, he ultimately published two comprehensive books, PiHKAL^46^ and TiHKAL,^29^ describing the chemicals he synthesized and their effects.

Dr. Nichols was engaged in studies to gain insights into how drugs interact with their target receptor to influence behaviors. Over the course of his career at Purdue University, Dr. Nichols also created hundreds of novel psychedelics based on all three known scaffolds. Each of these was designed with a purpose in mind, to help map key residues in the orthosteric binding pocket to chemical constituents on the ligand in order to generate a greater understanding of mechanisms underlying GPCR-ligand interactions.^47–49^ Shulgin and Nichols first published together in 1976 and were collaborators for several years.^50^ It is largely the effort of these two individuals that laid much of the foundation for modern psychedelic drug discovery and next-generation psychedelics.

Another chemist who played a prominent role in the early chemistry of psychedelics is Dr. Richard Glennon, who spent his career at Virginia Commonwealth University. In addition to synthesizing many new 5-HT_2A_ ^51^ ligands, he published in 1984 a study demonstrating that the affinity of ligands for the 5-HT_2_ receptor had a strong positive correlation with behavioral effects, thus demonstrating that the receptor target of psychedelics was the 5-HT_2_ receptor.^52^

Today there are a plethora of entities, both private and public, involved in psychedelic drug discovery (Figure 3).^53^ On the public academic front, there are research groups designing and testing new agonists to test specific hypotheses of drug/receptor interactions and effects on downstream signaling and behaviors, as well as designing synthetic schemes to generate new chemical entities (NCEs), all for the pursuit of fundamental knowledge. In the private sector, the efforts are focused on new routes and formulations of existing psychedelics as well as the development of NCEs to generate intellectual property (IP) that allow for the advancement of therapies that benefit the lives of patients within the context of the care model in the United States and peer societies.

**Figure 3:**
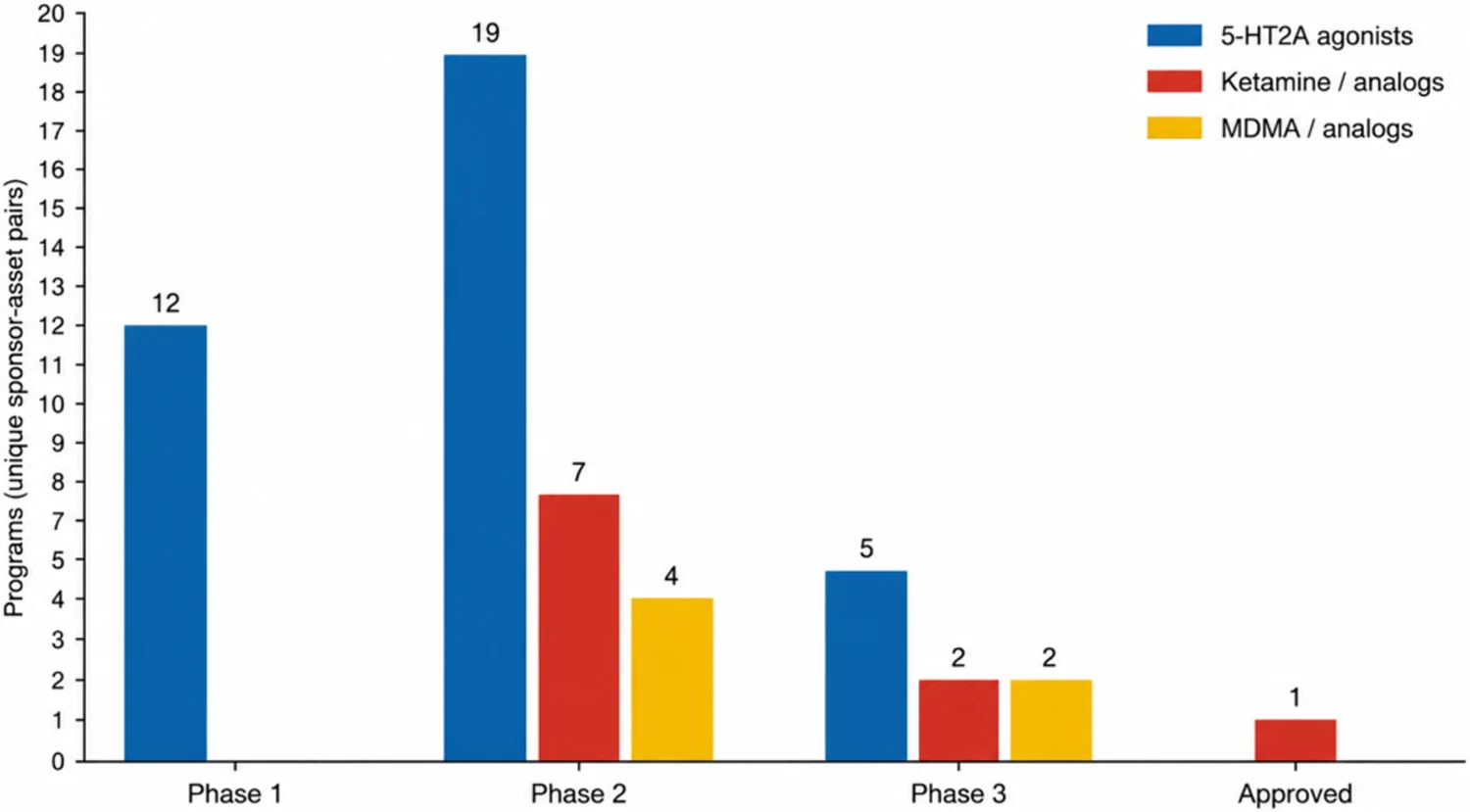
Psychedelic drug discovery has gained popularity in recent years. The number of psychedelic-related compounds/programs in various phases of clinical development. Data collected from Psychedelic α in August 2026.

Unfortunately, despite some recent requests for proposals, the federal governments of many countries, including the United States, have directly limited resources for funding research that would result in the discovery of new psychedelic molecules. Schedule I licenses are required for research with most psychedelics and apply to both the small quantities required for basic studies and the appreciably larger amounts needed for clinical trials. Though Schedule I compounds are defined as drugs, substances, or chemicals with no currently accepted medical use and a high potential for abuse, psychedelics remain highly restricted substances, even for research. Several patient and research advocacy organizations have attempted to work with both the FDA and DEA toward rescheduling of psychedelics for research purposes, but this has not yet been effective. Recent positive results in phase 3 trials of MDMA-assisted therapy for PTSD, despite recent FDA concerns related to inadequate controls, may create new support for rescheduling, and this will have a positive effect for research. Finally, the Federal Analog Act of the United States can subject certain novel psychedelic compounds that are substantially similar to schedule I or II substances to schedule I treatment when they are intended for human consumption.^54^

With the now widespread acknowledgment and acceptance of the potential of psychedelics as viable therapeutics for several conditions, there is now a tremendous financial incentive to develop new intellectually protected molecules with blockbuster potential. Due to the current lack of government funding, nearly every academic lab involved in psychedelic drug discovery has partnered with a private entity. The promise of these partnerships is funding for basic research and discovery through licensing agreements or research contracts, with the sponsor often having first right of refusal or exclusive rights to any IP resulting from that work, including NCEs.

Although ideal next-generation molecules for therapeutics may already exist within the hundreds of 5-HT_2A_ receptor agonists already described in the scientific literature, they are in the public domain, and there is not much if any IP that can be ascribed to them. It costs upward of one billion US dollars or more to bring a drug to market,^55^ therefore realistically, any new psychedelic brought through the development pipeline must have IP strongly associated with it. As major pharmaceutical companies have largely moved away from internal Research and Development, therapeutic advancement increasingly relies on acquisitions of assets that have been developed by smaller entities that have shown promise in early clinical efficacy trials. As of this writing, no major pharmaceutical company has fully embraced psychedelic drug discovery campaigns to identify novel molecules with their own internal program. Rather, large companies have recently acquired psychedelic drug assets for further clinical development by purchasing smaller startups that have demonstrated therapeutic efficacy in Phase II clinical trials. These include AbbVie’s purchase of Gilgamesh for bretisilocin, Otsuka’s purchase of Transcend for methylone, and Eli Lilly’s purchase of AtaiBeckley for 5-MeO–DMT.^56–58^ The amount of each purchase has been, remarkably, approximately in the $1–3 billion range. These purchases underscore the enthusiasm and potential for this new class of therapeutic.

Academic or industrial aside, the development of novel psychedelics can essentially be grouped into one of three distinct strategies. One involves unbiased high-throughput screening (HTS), which has been the traditional workhorse of lead identification in the pharmaceutical industry. Either by brute force or virtual screening, real or virtual ligands are screened against 5-HT_2A_ receptors in cellular functional assays or virtual docking *in silico* by the millions, if not billions. This is an expensive endeavor and is only employed by a few entities.^59^ Another involves creating libraries of psychedelic analogues using a function-oriented synthesis approach and assessing the effects on cell structure and function, rather than receptor engagement directly.^60,61^ A third method, which seems to be employed by most entities, is to slightly modify existing known psychedelics with known efficacy to a somewhat different molecule with IP associated with it. For example, Reunion Neuroscience is developing a prodrug of the known psychedelic 4-OH-DIPT,^62^ and Helus is developing deuterated psilocin.^63^ A fourth method is rational drug design, where analysis of the orthosteric binding pocket of the 5-HT_2A_ receptor is performed *in silico* and the findings are used to design NCEs that engage certain amino acid residues in perhaps a different way than known psychedelics.

Drug development in the psychedelic space can also be grouped into those developing fully behavioral NCEs and those developing nonpsychoactive analogs of psychedelics. In one camp are those that believe that the subjective trip is necessary for therapeutic benefit, and in the other, those that think the two may be dissociated. Within the former, there are efforts to develop NCEs with similar behavioral effects and duration to psilocybin but with strong IP associated with them. There are also efforts to develop short-acting psychedelics under the theory that it is not the duration of the subjective experience *per se* that provides therapeutic efficacy but just getting the patient to a peak subjective experience for a minimal time that provides efficacy. For example, the primary asset Eli Lilly acquired from AtaiBeckley is a novel formulation of a classic psychedelic: intranasal 5-MeO–DMT. Amandala Neuro, a spin-out of AtaiBeckley, is developing a short-acting intravenous formulation of psilocin that has shown therapeutic efficacy in a Phase II trial for major depression.^64^

As we are beginning to understand the receptor pharmacology of psychedelics better from SAR studies,^65,66^ this has allowed for the possibility of nonpsychoactive agonists for 5-HT_2A_ receptors, as discussed above. It remains to be seen, however, if nonpsychoactive 5-HT_2A_ receptor agonists will demonstrate therapeutic efficacy in larger Phase III clinical trials and eventually come to market.

### Mechanism of Action

Though the development of NCEs is an active and heavily funded area of research, little is known about their therapeutic mechanism of action, particularly for nonpsychoactive analogues. More research has been done to determine the mechanism of action of classic psychedelic compounds, which may prove to be useful in understanding the actions of any derivatives.

Without doubt, the 5-HT_2A_ receptor plays an integral role in the psychedelic experience.^67–69^ Many arguing that the psychedelic experience is deeply necessary for the therapeutic effects would therefore argue that 5-HT_2A_ is also a therapeutic target, and the two cannot be separated. For scientists trying to disentangle the hallucinogenic and therapeutic effects through development of nonpsychedelic analogues, the role of the 5-HT_2A_ receptor in mediating therapeutic responses remains controversial. Further complicating the matter, rodents have a polymorphism in the orthosteric binding pocket of 5-HT_2A_, which results in different dissociation rates for tryptamine compounds compared to the human 5-HT_2A_.^70,71^ Nonhuman primates, however, have nearly identical 5-HT_2A_ sequences to humans (NP_001028138.1, NP_000612.1). Finally, the 5-HT_2A_ agonist lisuride is not hallucinogenic.^72^

There have been several hypotheses put forward about the therapeutic mechanisms of action of psychedelics. One hypothesis posits that although the hallucinogenic aspects of the drug are mediated by the 5-HT_2A_ receptor, the therapeutic actions of psychedelics can be mediated through activation of a different receptor: likely 5-HT_2C_ or direct binding to TrkB receptors.^73,74^ Recent evidence has failed to replicate the TrkB findings.^45^ 5-HT_1A_ and TAAR have also been implicated in psychedelic actions.^75–78^ The notion of multiple receptor involvement is supported through findings that suggest that 5-HT_2A_ antagonism is insufficient for blocking the therapeutic effects of psychedelic compounds,^79^ although this remains to be validated.

On the other hand, there is more evidence to suggest that the engagement of 5-HT_2A_ is necessary to achieve therapeutic effects. Studies using the 5-HT_2A_ receptor antagonist ketanserin are sufficient for blocking psychedelic-induced growth of cortical neurons^19,21,61^ as well as various behaviors reflective of antidepressant efficacy or other therapeutic effects.^80–82^ However, ketanserin is not selective between 5-HT_2_ receptors in rodents, and it inhibits other receptors involved in neural function (such as histaminergic and adrenergic systems), leaving open the possibility of involvement of 5-HT_2C_ or other targets. A few studies have demonstrated the necessity of 5-HT_2A_ involvement in cortical neuronal growth and therapeutic responses using a more specific antagonist, M100907 (volanserin).^66,83–85^

Recently, endeavors using genetic deletion of 5-HT_2A_ receptors in rodents have illustrated the necessity of this receptor in psychedelic-mediated fear extinction learning, drug extinction learning, as well as motivated behaviors (in tail suspension test and sucrose preference test), all of which depend on engagement with 5-HT_2A_.^80,81,86^ Psychedelic-mediated dendritogenesis and spinogenesis are also blocked in these animal models, suggesting that engagement of 5-HT_2A_ is necessary.^21,81^ Recent work has suggested that activation of the intracellular pool of 5-HT_2A_ receptors may be important for the action of some psychedelic drugs.^21^

Initial studies with nonpsychoactive analogues suggest that engagement of 5-HT_2A_ receptor activity is also necessary. Antagonist administration (ketanserin or M100907) is sufficient for blocking the therapeutic behavioral actions of nonpsychoactive compounds,^60,87^ although off-target effects cannot be ruled out due to the nature of ketanserin. More recently, Aarrestad et al. demonstrated that a third-generation, nonpsychoactive analogue of a psychedelic, tabernanthalog, is ineffective when administered to 5-HT_2A_-KO animals.^88^

If action at the 5-HT_2A_ receptor is necessary, there are a few mechanisms by which agonists can target the same receptor but have different downstream effects (i.e., hallucinogenic vs not). The first theory is functional selectivity; the idea that different agonists cause different conformational changes to the receptor through differential interactions with amino acid residues in the orthosteric binding pocket of the receptor, thereby eliciting different downstream effects. Current evidence suggests that psychedelics and novel analogues primarily signal through Gq pathways,^45^ though this has yet to be fully tested. Interestingly, the on/off kinetics of various compounds at 5-HT_2A_ receptors do not predict hallucinogenic properties.^45^ A second theory involves a different type of functional selectivity, wherein binding of ligands allows signaling through a dimer complex of 5-HT_2A_ and mGluR2. Work by Gonzalez-Maeso and colleagues has elegantly demonstrated signaling through this dimer when stimulated with psychedelic compounds, but not antipsychotic agents.^89^ Psychedelics may signal through this dimer interaction, though nonhallucinogenic analogues do not; this hypothesis has yet to be tested. Finally, a paper by the Kim lab has demonstrated that stimulating a psychedelic-responsive ensemble in the mPFC elicits anxiolytic properties without a head-twitch response behavior, suggesting there are different brain regions responsible for therapeutic versus hallucinatory effects of these compounds.^90^ From a drug discovery standpoint, it is possible that the 5-HT_2A_ receptor responds differently to psychedelic compounds depending on proteins in its immediate environment. This has been documented for several receptor/G-protein pairs based on their localization in the membrane microdomains (i.e., lipid rafts).^91,92^ This could be via direct or indirect (downstream) interactions after 5-HT_2A_ receptor activation. For example, if hallucinatory effects function through receptor dimerization, perhaps this only occurs in some brain regions (i.e., claustrum) but not others (i.e., mPFC). In this way, nonpsychoactive analogues of psychedelics may be able to target these distinct differences or engage downstream targets. The differences in 5-HT_2A_ activation across brain regions are an important area of research for elucidating optimal drug targets.

Though necessary, nonpsychoactive psychedelic analogues can have decreased activity as measured by canonical Gq signaling downstream of 5-HT_2A_ receptors compared to their psychedelic parent compounds. Given that 5-HT_2A_ is the most highly expressed excitatory 5-HT receptor in the brain,^93–96^ it is possible that even with reduced activity, the sheer number of receptors activated is enough to elicit a therapeutic response.

One hypothesis that needs to be rigorously tested is whether nonpsychoactive analogs are just “weaker psychedelics.” A sufficient amount of 5-HT_2A_ activation may be achieved to elicit therapeutic responses (cortical neuron growth, increased behavioral motivation), without eliciting perceptual effects. A recent study by Wallach et al. concludes that an activation of 70% or greater at the Gq pathway is necessary for behavioral effects.^65^ This work was conducted in immortalized cell lines and should be further investigated in neurons. In unpublished work, pilot attempts to answer this question in SH-SY5Y cells have yielded similar but differing results.^97^ In the same vein, microdosing—taking small doses of psychedelic substances to theoretically support focus, executive control, and goal-directed behavior, which may result in secondary improvements in emotional regulation and psychiatric wellbeing—would fall into this category. The bulk of microdosing research appears to be inconclusive and/or poorly blinded. The minimum level of cortical or subcortical activation required to elicit growth without perceptual effects has yet to be tested directly.

Finally, a circuit-level view is necessary for assessing the true therapeutic potential of these compounds. While the 5-HT_2A_ receptor appears important, all psychedelics and nonpsychoactive analogues studied today engage a plethora of targets.^45^ The summation of receptor activation and what downstream circuits may be indirectly activated for therapeutic and/or perceptual effects requires further study.

While nonpsychoactive analogues hold promise for bringing a more accessible medication to the public, it is possible that hallucinations provide a more robust or more long-lasting therapeutic effect.

### Animal Models

Animal models are a critical component of drug discovery and development. Such models offer advantages for defining psychedelics’ mechanism of action, neurobiological mechanisms such as neuroplasticity, duration of action, and biomarkers. These models may help to unlock the capacity to develop the next generation of psychedelics. There are notable advantages of animal models, such as rigorous control of experimental variables, the capacity to perform deep mechanistic studies, the capacity to use sophisticated genetic and neural circuitry techniques, the capacity to perform longitudinal studies throughout the lifespan of the organism, and the capacity to validate and extend cellular findings and mechanisms. For example, it is well established that the history of the organism (including behavioral and drug history) and the current environment in which a drug is administered can influence the effects of the administered drug.^98,99^ This is certainly true for the psychedelics, and has been referred to as “set and setting.” ^14,93,100^ Laboratory animals provide exquisite control over the history of the organism and its current environment. In combination with rigorous techniques from the experimental analysis of behavior, this can provide important insights into the in vivo pharmacology of psychedelics.

Animal models can be used to define the fundamental pharmacology of psilocybin. This is an excellent pairing with in vitro and cellular assays. Concordance between cellular and laboratory animal pharmacology end points supports the rationale that findings will generalize to humans, including in clinical settings. It is also important to note that several influential ideas in the psychedelic field, such as biased agonism and intracellular receptor signaling that especially relate to the development of next-generation psychedelics, have garnered disproportionate support from cellular assays that could be susceptible to artificial cellular conditions that may not occur in whole organisms. Additional support from in vivo pharmacology experiments would strengthen the case that such ideas will have therapeutic relevance.

Historically, the bulk of animal research with psychedelics has involved studying their fundamental in vivo and behavioral pharmacology, especially to delineate the receptors that mediate specific behavioral responses, shared effects between different psychedelics and among different classes of psychedelics, and to define pharmacological changes such as tolerance. Almost no research has focused on therapeutic applications. This is because many of the animal studies with psychedelics that occurred between the 1970s and early 2000s focused on their misuse liability. During that period, scientifically, the psychedelic effect itself was considered to be a marker of misuse liability. More practically, during that time, the limited available funding for research with psychedelics was issued for the study of their harm. Despite these constraints, research from the Nichols, Glennon, Sanders-Bush, Winter, Cunningham, Howell, and Fantegrossi laboratories, among others, did pioneering research into the behavioral pharmacology and neuropharmacology of psychedelics. With the renaissance of psychedelic research that has occurred in the past 15 years or so, and especially the profound therapeutic responses that have been reported in clinical research, there is a new impetus to generate animal models that would capture and allow for a deep mechanistic understanding of the therapeutic response to psychedelics.

There are three major categories of behavioral pharmacology techniques that are commonly applied across different classes of psychoactive drugs: assays of the directly elicited effects of drugs, assays of their interoceptive/subjective effects, and assays of their hedonic, reinforcing, or motivational effects. This largely parallels the assays that can be used in defining the misuse liability and addictive potential of substances in humans. Studying directly elicited effects can be used to study the in vivo pharmacology of psychoactive drugs^101^ by the measurement of an unconditioned behavior following drug administration. Such procedures have been applied to stimulants using locomotor activity, opioids with the Straub tail response, and cannabinoids with the tetrad procedure. In the context of psychedelics, the unconditioned behavior that has been most commonly studied is the head-twitch response (HTR).^102,103^ The HTR is commonly used to study effects of psychedelics in rodents and presents as rapid, rotational jerks of the head or high-frequency paroxysmal head rotation.^104^ Head twitching occurs in rodents spontaneously but, importantly, is selectively increased in frequency by psychedelics.^105–110^ The number of head twitches in a given period of time is correlated to psychedelic potency in humans.^111^ This assay has been widely used by behavioral pharmacologists for decades and has provided critical data demonstrating that serotonin 5-HT_2A_ receptors are a critical molecular mediator of this elicited response, as the HTR is blocked by the selective antagonist M100907 ^112,113^ or genetic ablation of the 5-HT_2A_ receptor.^112–115^ Likewise, it has been suggested that findings from this assay can shed light on the mechanisms underlying the psychoactive effects of psychedelics. This idea rests primarily on the correlations of potency antagonist blockade of the HTR with affinity for 5-HT_2A_ receptors,^110,116^ affinity at 5-HT_2_ receptors, and psychedelic potency in humans.^117^ Furthermore, this assay may have utility in the development of the next generation of psychedelics, as there is interest in the idea that compounds that lack psychedelic activity can maintain the therapeutic response. As such, a positive response in a therapeutic assay concurrent with a negative response in the HTR assay would possibly indicate a compound with the potential to treat addiction, depression, anxiety, or other disease in the absence of psychedelic activity. Finally, this assay continues to have utility in defining fundamental behavioral, pharmacological, and neuropharmacological mechanisms. The HTR appears to have the capacity to discriminate psychedelic from nonpsychoactive drugs that both act as agonists of 5-HT_2A_ receptors.

While the HTR has provided enormous utility in psychedelic research, especially in their behavioral pharmacology and receptor mechanisms, it is important to consider the nuances of this assay and that, like any assay, it has limitations on its application and interpretation. For one, it is important to note that there are many other unconditioned effects elicited by psychedelics and that the presentation of such elicited responses can vary among species. Psychedelics can also elicit changes in social behavior, induce convulsions and seizures at higher doses,^31^ and exhibit other elicited effects if they have polypharmacological activity at a diversity of serotonin receptors, particularly the 5-HT_1A_ and 5-HT_2C_ receptors. The HTR is typically measured in mice but has a more whole-body presentation in rats, where it is described as wet dog shakes.^104^ Nonhuman primates do not exhibit either response but rather exhibit a psychedelic-elicited fly-catching behavior. Similarly, there is no established human counterpart to the HTR. Second, there are several notable false positives with this assay, in that there are 5-HT agonists that produce HTR but do not produce psychedelic subjective effects in humans.^117,118^ Third, the HTR does not seem to be a particular quantitative measure of psychedelic activity in that there are strong psychedelics that produce a weaker HTR (notably the tryptamine analogues) and strong psychedelics that produce a more robust HTR (notably the phenethylamine analogues). As such, there may be a threshold level, rather than a linear level, of the HTR that defines a psychedelic response, but this remains to be determined. Finally, it is possible that the HTR involves elements of spinal reflexes rather than brain-mediated responses, which could have great meaning when species differences in the 5-HT2A receptor and its distribution are considered. Nonetheless, HTR remains a widely used assay of target engagement of 5-HT2A receptors in whole organisms, in part because this unconditioned behavior can be assessed without any training or other extensive procedures, as is the case, for example, with drug discrimination.

An alternative to the HTR is drug discrimination assays. For several decades, these were mainstays in the study of psychedelics but for the past decade or so have fallen out of favor. Drug discrimination is a complex learning task in which an animal learns to press one lever when it receives the training drug and another lever when it receives a vehicle. Responding on the correct lever produces the presentation of a food reinforcer such as a sucrose pellet. The animal is trained to discriminate the interoceptive effects of a psychedelic, typically in comparison to vehicle, by associating these interoceptive effects with responding on the drug-paired lever.^119,120^ Specifically, on sessions when the animal has received pretreatment with a specific drug, a particular response in the chamber (e.g., left lever presses) is reinforced (e.g., with food). On other days when the animal has received vehicle pretreatment, another particular response (e.g., right lever presses) is reinforced. After a number of training sessions, the animal can discriminate the test drug with high reliability (e.g., with ≥ 90% accuracy). While there is complexity to this, these interoceptive effects are often thought to be analogous to the subjective effects produced in humans, such as psychedelic activity.

However, human research using drug discrimination has suggested that drug discrimination and subjective effects are not identical but rather provide complementary results.^118^ Upon successful training, the animal can be exposed to test drugs to determine if they produce interoceptive effects that are similar in potency and/or efficacy to those of the training drug. This is a powerful assay in that it allows the experimenter to establish key aspects of the subjective and psychoactive effects of psychedelics, including their relationship to therapeutic responses and underlying mechanisms. As there has been great debate in the clinical literature regarding the role of subjective effects in therapeutic response, this assay could be pivotal in the development of the next generation of psychedelics. In addition, drug discrimination also provides a measure of the rate of response, which can be used as an internal control to rigorously verify that a physiologically and pharmacologically relevant dose range is being tested. Drug discrimination can also provide information on the onset and duration of action of the subjective experience of a psychedelic, something with profound implications for the access and scalability of next-generation psychedelics.^121^ As such, drug discrimination provides notable advantages over the HTR in the specific modeling of the interoceptive/subjective effects of psychedelics.^122^ This assay allows the experimenter to recapitulate drug responses (e.g., potency and efficacy) and doses that are characteristic of the psychedelic response in humans. Given the low throughput of this assay, it has yet to be extensively used to test novel nonpsychoactive analogues of psychedelic compounds. We encourage authors entering this field to use this assay or variants when possible.

Drug self-administration is the gold standard for studying the reinforcing and addictive effects of hedonic drugs (like opioids). Self-administration specifically examines the extent to which a drug of misuse serves as an operant reinforcer. This can be done with a design in which an operant response such as a lever press or a nose poke occasions an injection of drug through a diversity of routes of administration, including intravenous, oral, inhaled, or other routes. The most common route is intravenous administration, as this facilitates the capacity of the animal to learn the association between the response and the initiation of the drug effect. Drug self-administration assays can result in multiple meaningful metrics of drug reinforcement, including overall drug consumption, response rate, breakpoint, or several behavioral economic measures, including demand intensity and demand elasticity for the drug.^123,124^

Self-administration models can also be embedded in reinstatement experimental methods, which test the ability of a drug-associated cue or context to once again prompt drug self-administration behavior after a period in which self-administration of the drug has been extinguished.^125^ During much of the 20th and early 21st centuries, the use of self-administration procedures with psychedelics was focused on establishing their potential misuse liability and addiction potential. The consistent finding from this research is that animals will not readily self-administer psychedelics.^82,93,119,120,126^ This lies in stark contrast to nearly every other class of misused or addictive substances, including alcohol, stimulants, sedatives, nicotine, and cannabinoids, all of which show robust self-administration under appropriate conditions. Indeed, part of the reason that self-administration is considered a gold standard assay is the finding of a nearly perfect alignment between which substances are misused by humans and self-administered by animals, with the notable and sole exception of psychedelics.

Generally, reinforcing properties of drugs are thought to be mediated by dopamine (DA) increases in key areas of the brain such as the nucleus accumbens (NA) and ventral tegmental area (VTA). All strongly reinforcing drugs increase dopamine levels in the NA directly or indirectly. In contrast, there is very little evidence that psychedelics can produce such effects. There have been several preliminary efforts to create procedures that allow for psychedelic self-administration, using agents such as LSD, DMT, psilocybin, mescaline, and DOI, all of which have failed to produce even one consistently self-administering animal.^82,119,126–129^ In one study, a single rhesus monkey did reliably self-administer DMT, but only after 6 days of complete sensory deprivation.^130^ It is well established that a drug history in animals trained to self-administer drugs is a critical determinant in whether and to what extent these animals will self-administer different drugs. This is especially the case for self-administration of a drug by an animal experienced with or maintained on a drug with similar interoceptive effects.^131,132^ Even under conditions when drugs are substituted for maintenance drugs with similar discriminative stimulus/interoceptive properties such as MDMA,^105^ it is difficult to document self-administration reliably. However, even though self-administration is not maintained for any extended period of time, there were several experimental sessions that occurred with no apparent pattern in which monkeys would earn most or all of the available injections of DMT, psilocybin, or mescaline when substituted for MDMA at rates of responding several times that of the saline control, which is consistent with use patterns in humans.

Following the renaissance in psychedelic research and the new impetus to study their therapeutic properties, increased examination of psychedelic compounds in their potential to change the self-administration of addictive drugs would significantly propel the area of psychedelics for substance use disorders. Very few studies have been published in this regard. Only recently, two groups published that the psychedelic DOI attenuates the reinforcing effects of alcohol.^85,133^ To our knowledge, these were the very first studies to report that a psychedelic can attenuate the self-administration of any misused drug.

As mentioned, there is now a new impetus to research on the therapeutic mechanisms of psychedelics. There are several caveats in this regard. For one, it is not yet settled whether the therapeutic response to psychedelics relies on the induction of a mystical experience. If such experiences turn out to be necessary, it is unclear whether there is any animal analog to such an effect or, if it occurs, how it can be observed, quantified, and measured by an experimenter. One of the effects of psychedelics in humans is ineffability, meaning that the experience surpasses the capacity of language to express it. One of the long-standing limitations of animal models is the need to measure and interpret effects in a nonverbal species. This may be even more challenging, given that even a verbal species has difficulty conveying what occurred. Second, the clinically measured therapeutic responses to psychedelics are remarkable in a number of ways, including that under optimized, prepared, and supportive conditions,^40^ they occur in a large portion of participants, they are very strong effects, they occur seemingly instantly, they occur in individuals with long-standing and intractable illnesses, and they are persistent following a single administration. Some of these findings will make it easier to study the underlying mechanisms in animals, including that these are robust effects that emerge quickly. Some will make it more challenging, such as their persistence and that they may emerge following long illness histories. Third, it is believed that the “set and setting” strongly influence the therapeutic responses, including elements of preparation and supportive psychotherapy.

This may be very challenging to translate into animals, though attempts have been made.^134^ To date, no published studies have reported attempts to use procedures such as ‘preparation’ in animal models. Likewise, it is unclear whether setting elements such as specific music or lighting conditions would attenuate or augment the therapeutic response in animals. Overcoming some of these challenges will require exciting and rigorous research, and success would greatly aid in the development of the next generation of psychedelic drug development.

As with behavioral pharmacology, there are many well-validated procedures that have been used for decades in the field of behavioral neuroscience. The forced swim test (FST) and tail suspension test (TST) are quick tests that are used to test certain behaviors in mice and rats.^135–137^ For the FST, animals are placed in a tank of room temperature to lukewarm water such that they can not touch the bottom with either their paws or tails and are observed for 5–6 min. During this time, the animal will either move about (e.g., swimming, climbing; motivated behaviors) or float immobile (‘give up’, behavioral despair). Various forms of stressors have been shown to lead to increased immobility, and presumably behavioral despair, and treatment with certain antidepressants like SSRIs can reverse this stress-induced immobility. Similarly, the TST suspends a mouse up by its tail for 6 min, and the amount of time the animal spends moving and trying to escape during the last 4 min is quantified as motivated behavior. Certain stress paradigms result in more immobility and simple hanging in this assay. These tests have good predictive validity for identifying drugs with antidepressant efficacy in humans. Several research groups have demonstrated that both psychedelics and nonpsychoactive analogs of psychedelics are efficacious at restoring motivated behaviors in these paradigms in both mice and rats.^20,21,60,81,88^ There are unfortunately two inherent problems with FST, one of which is shared with TST. For both FST and TST, drug dosing is typically subchronic, with dosing 24, 5, and 1 h prior to testing to cause reversal of the “depressed” phenotypic response.^138^ However, these agents (e.g., SSRIs, tricyclic antidepressants) show a delayed (up to 2 months) therapeutic onset in humans. This is in contrast to psychedelics, which can produce a rapid reversal of the “depressed” phenotype that can last for months after a single administration. Additionally, in the FST, rodents can learn that if they float without having to struggle (passive coping mechanism), they will be rescued, so repeated use of FST in an individual animal may test learning as much as “depression”.

The Sucrose Preference Test (SPT) is used as a measure of anhedonia in rodents.^139^ When an animal is presented with two bottles (one with regular water, and the other sweetened with sucrose), the animal will prefer sucrose water. After chronic stress and/or induction of a depressive phenotype, the animal loses its preference for the rewarding bottle. This is seen as anhedonic response. As with FST and TST, this can be reversed with an antidepressant treatment. Multiple groups have demonstrated that after chronic stress, sucrose preference declines and psilocybin rescues this phenotype.^79,81^

Learned helplessness has excellent face value and predictive value.^140^ Briefly, an animal is subjected to an inescapable stressor (e.g electric shocks), which induces a depressive-like state such that, when given an exit strategy, animals “endure” the stressor rather than trying to escape (learned helplessness). “Giving up” is a common symptom in patients with depression. The Kwan lab has demonstrated that psilocybin has the potential to reverse this behavior and restore motivated behaviors.^18,86^

Another test that has been used to assess the behavioral effects of psilocybin is object-pattern separation.^141^ This is a cognitive behavior dependent on a specific hippocampal circuit shared between rodents and humans and therefore has high translational value. Chronic stress in rats eliminates the ability of the animals to detect pattern changes in their environment, and a single treatment with psilocybin appears to reverse the effects of stress long-term and restore the ability to recognize pattern changes.^142^

### Biomarkers

Great progress has been made in establishing possible benefits from psychedelic therapy for mood disorders, but there is still much to be done. Progress depends on several factors that currently influence successful or useful outcomes for both basic and clinical research.

There are two related issues that are not unique to psychedelics, but are certainly vexing to their development as potential therapeutics. One concerns current “quantification” of the symptoms of mood and anxiety disorders and the quantification of the extent to which they contribute to the degree of disability in a given subject. For the issue of disease/disability quantification, there is no shortage of rating scales that create a profile of disease within a given subject (depression and anxiety are most often comorbid) and assign a numerical value. Some of these tests are self-administered and others are administered by a professional (not necessarily one with extensive mental health experience). All of these scales are “fuzzy” and dependent on the current mindset of the rater (whether self-administered or not). Another notable exception for these concerns is substance use disorders, where biological confirmation of drug use status at study intake and drug use or abstinence after treatment substantially addresses those concerns. One example is research using psilocybin to treat tobacco use disorder, in which exhaled carbon monoxide and urinary cotinine are used as methods for bioverification of tobacco use vs abstinence.^6^ Another example has been research using psilocybin to treat alcohol use disorder, where hair or fingernail samples have been analyzed for ethylglucuronide concentration as bioverification of abstinence self-report in a subset of participants.^5^ However, there are no such biomarkers for psychiatric disorders outside of substance use. Thus, it is imperative to establish biological hallmarks (biomarkers) that can be used to both confirm and quantify diagnosis. As of this writing, this has proven elusive. A profound limitation of current research is the lack of biomarkers for psychedelic therapeutic activity in early drug development. This is important because many of the limitations of first-generation compounds listed above relate to properties (such as the nature of subjective experience) that are indirectly tied to therapeutics and which are lacking biomarkers.

One significant problem for biological hallmarks of disease is that at least in the case of major depressive disorder, what is described as a single entity may have a number of root causes that manifest as a constellation of symptoms. Genomics have not proven particularly useful for understanding depression, and some of the genes thought to cause depression-like behaviors in mice have not been observed to be relevant for depression in humans.^143^ Two published studies examined major depressive disorder proteomes—deduced from genome/gene expression^144^ and mRNA for prediction of mood state and treatment response.^145^ There was no overlap in targets between these studies, illustrating the difficulty of genetic/genomic accounting in the assignment of a biomarker.

Imaging—including EEG, fMRI, and DTI—has been used to indicate both depression and likely treatment response,^146–149^ and there are recent suggestions that structural brain imaging might predict treatment response for depression.^150^ However, it is important to recognize that “prediction” in such studies will refer to the observation of group differences based on disorder or prepost analysis. Advances will be needed in order to have greater predictability at the individual level for either diagnosis or clinical prediction of treatment response. Analysis of digital data obtained from cellphone keyboards (keystrokes, mistakes) may also prove as a biomarker for mood.^151^

One consistent hallmark of depression, in both human and animal models, has been attenuation of cAMP signaling, which is restored upon successful antidepressant response. This has been observed in 11C Rolipram PET imaging studies, where brain cAMP was lower during depression and returned to baseline in those subjects showing positive clinical response to antidepressant therapy.^152^ Recently, this has been translated into a simple platelet biomarker, wherein augmentation of the activation of the cAMP-generating enzyme, adenylyl cyclase, by the G protein (Gsα), reveals the presence of depression and tracks with positive antidepressant responses.^153,154^ It is hoped that these biomarkers, and those yet to be developed, show a continuity for depression diagnostics as well as a confluence of response to antidepressant agents, including psychedelics. A clear demonstration of common diagnostic and predictive pathways will certainly help to establish clinical validity and utility for psychedelic compounds.

### Critical Assays, Summary of Strengths/Weaknesses of the Field, Pathways Forward

The field of psychedelic science has expanded rapidly in recent decades and made enormous progress for treating neuropsychiatric disorders. While preliminary research with first-generation psychedelics is very promising, there are several limitations of these compounds and treatments that may limit their clinical dissemination. The surge of novel psychedelics and psychedelic analogues provides hope for improving patient care and expanding dissemination while minimizing side effects. Current animal behavioral studies are beneficial, though further refinement of assays like HTR and FST and the development of novel tests with greater sensitivity and reliability would help to push the field forward in screening novel compounds. Finally, biomarkers in humans may help to diagnose and guide the therapeutic treatment for patients.

Our work has summarized the efforts in psychedelic drug discovery, the hurdles, the triumphs, and possibilities. We hope that this serves to ground us as we push the boundaries of the field forward. Hopefully, the next few years will show great progress toward providing novel therapeutics for those who suffer.
